# Carbon dots: a safe nanoscale substance for the immunologic system of mice

**DOI:** 10.1186/1556-276X-8-276

**Published:** 2013-06-08

**Authors:** Zhongcai Gao, Guangxia Shen, Xiunan Zhao, Na Dong, Peiyuan Jia, Junhua Wu, Daxiang Cui, Yingge Zhang, Yuxia Wang

**Affiliations:** 1 Beijing Institute of Pharmacology and Toxicology, Beijing 100850, People’s Republic of China; 2 National Key Laboratory of Nano/Micro Fabrication Technology, Research Institute of Micro/Nano Science and Technology, Shanghai Jiao Tong University, Shanghai 200240, People’s Republic of China

**Keywords:** Carbon dots, Immune function, Splenocyte proliferation, Cytokine

## Abstract

We aimed at investigating the effect of carbon dots on the BALB/c mice immune system. Mice were respectively treated with different doses of carbon dots and saline. At 1 and 9 days after intravenous administration of carbon dots, splenocyte proliferation, subpopulation of the peripheral lymphocytes, and induction of primary immune responses in mice were investigated. The results showed that high dose of carbon dots could promote the percentages of CD3+ and interferon-γ (IFN-γ) secretion and decrease the proportions of CD4+/CD8+ on the first day after administration. At 9 days post exposure, the proliferation of splenocytes had a significant increase. IFN-γ secretion and proportions of CD3+/CD19+ were also found to have an obvious promotion, and both the percentages of CD4+ and CD8+ T lymphocytes were raised, whereas the expression of cytokines made little change in the treated groups, except for IL-12 which had a slight increase in the 50-mg/kg group. The weight coefficients and histological analysis of the spleen and thymus of the treated mice exerted fewer differences compared with those from the control mice. It suggests that carbon dots could influence the immune functions of normal BALB/c mice by inducing Th1 and Tc responses and that these effects were not enough to induce the morphological change of the immune organs.

## Background

Two-photon-fluorescent nanoparticles, primarily quantum dots (QDs), have recently attracted much attention for their many promising applications, especially in the field of biomedical imaging [1,2] and detection [3-5]. These QDs are considered as being more advantageous over conventional organic dyes in terms of optical brightness, photostability, and resistance to metabolic degradation [6]. However, heavy metals as the essential elements in QDs have prompted serious health and environmental concerns [7]. Therefore, the search for benign alternatives has become increasingly important and urgent. Sun et al. discovered that nanosized pure carbon particles may be surface-passivated to exhibit bright photoluminescence in the visible and near-infrared spectral regions [8]. These photoluminescent carbon nanoparticles, abbreviated as carbon dots, were found to be physicochemically and photochemically stable and non-blinking and exhibited very high two-photon absorption cross sections [9,10]. Carbon dots as a new class of QD-like fluorescent nanomaterials have been widely explored in biological applications and beyond [9-12].

Carbon has generally not been considered as a toxic element; however, there are growing evidence and controversies concerning the toxicity of fullerenes and carbon nanotubes [13-15]. For special material configurations and structures found in carbon dots, it is essential to evaluate their biocompatibility *in vitro* and *in vivo*. In this contribution, we investigated the effects of carbon dots on the immune function of normal BALB/c mice to elucidate the interactions between carbon dots and the immune system and to explore more theoretic evidence for the application of carbon dots in the field of medical diagnosis and biotherapeutics.

## Methods

### Experimental agents

Experimental agents were sourced from the following locations: raw soot (Jixi Kaiwen Hu Limited Co., Jixi, Anhui, China); RPMI-1640 (HyClone, Thermo Scientific Co., Waltham, MA, USA); anti-CD3 (peridinin-chlorophyll protein complex (Percp)), anti-CD19 (adenomatous polyposis coli (APC)), anti-CD4 (phycoerythrin (PE)), anti-CD8 (fluorescein isothiocyanate (FITC)) (Becton Dikinson Company, Franklin Lakes, NJ, USA); fetal bovine serum (FBS; HyClone, Thermo Scientific Co. USA); bicinchoninic acid (BCA) protein assay kit (Nanjing Keygen Biotech. Co., Ltd, China); interleukin-4 (IL-4) and interferon-γ (IFN-γ) enzyme-linked immunosorbent assay (ELISA) kit (Neobioscience Technology Co., Ltd, Shenzhen, China); concanavalin A (ConA), lipopolysaccharide (LPS) (Sigma-Aldrich Co., St Louis, MO, USA); anti-TNF-α, anti-IL-12, anti-IFN-γ, anti-IL-4 (Santa Cruz Biotechnology, Inc, Santa Cruz, CA, USA); rabbit anti-β-actin (Beijing Biosynthesis Biotechnology Co., Ltd, Beijing, China).

### Synthesis and characterization of carbon dots

Carbon dots were prepared using the improved nitric acid oxidation method. In the typical experiment, 0.5 g of raw soot was dispersed ultrasonically in acetone solution for 30 min and then centrifugated and dried under vacuum at 80°C. Subsequently, the cleaned soot was refluxed in 25-ml 5 M HNO_3_ at 120°C for 14 h. The black suspension was cooled down to room temperature and centrifugated at 3,000 rpm for 10 min to remove the unreacted precipitate. The light brown solution was neutralized by Na_2_CO_3_ and then dialyzed against Millipore pure water (Billerica, MA, USA) for 3 days to remove the salt of sodium through a dialysis membrane with an MW cutoff value of 1,000, affording purified carbon nanoparticles. After that, further size separation of carbon nanoparticles was performed by stepwise ultrafiltration with NMWL values of 5,000 and 3,000 ultrafiltration membranes using a Millipore stirred ultrafiltration cell. Finally, the carbon dots were dialyzed with an MCO of 3,000 dialysis membrane.

Atomic force microscopy (AFM) images of carbon dots were taken on a MultiMode Nanoscope III, a scanning probe microscopy system (Veeco, Plainview, NY, USA). The samples for AFM were prepared by dropping an aqueous suspension (0.01 mg/ml) of carbon dots on freshly cleaved mica surface and dried under vacuum at 80°C. UV-visible (vis) spectra were measured at 20°C with a Shimadzu UV-2450 UV–vis spectrophotometer (Kyoto, Japan) equipped with a 10-mm quartz cell, where the light path length was 1 cm. Fluorescence spectra were recorded on a HITACHI H FL-4600 spectrofluorimeter (Tokyo Japan).

### Animal injection, weight measurements, and sample collection

Female BALB/c mice (approximately 18 to 22 g), obtained from the Center of Laboratory Animals of Academy of Military Medical Sciences in compliance with the Institutional Animal Care and Use Program Guidelines, were given food and water *ad libitum* and housed in a 12-h/12-h light/dark cycle. After acclimation, the mice were randomly divided into eight experimental groups, each consisting of ten mice. Before intravenous administration to mice, the carbon dots were well sonicated and diluted in physiologic saline. The six groups for administration were injected in the tail vein with carbon dots at 2, 10, and 50 mg/kg (each dose is treated to two groups), and the last two groups were treated with saline as control. At 1 and 9 days post exposure, body weights of the mice were measured. Thereafter, the blood samples were collected and the mice were sacrificed. Spleen and thymus samples were surgically removed immediately and weighed in a sterile hood. One part of organ samples was cut off and fixed in 4% formaldehyde solution, and the other parts were used for immunological assays. The weight coefficients of the spleen or thymus (%) = spleen or thymus weight (g)/mice body weight (g) × 100. Blood samples obtained from the mice were centrifuged (12,000 rpm) for 10 min at 4°C to separate serum and blood cells. The serum was stored at −80°C for determination of cytokines. For histopathological observation, the thin-sectioned tissue specimens were stained with hematoxylin and eosin and examined under light microscopy.

### Lymphocyte proliferation assay

Single-cell suspensions were prepared from the spleens in RPMI-1640 medium. Firstly, fresh spleens (*n* = 5 per group) were put into 5 ml of RPMI-1640 before grinding the organs with a syringe core on the nylon net (200 meshes) to prepare crude splenocyte suspension. The suspension was freed from debris by centrifugation at 1,000 rpm for 10 min at 4°C. The remaining splenocyte suspension was resuspended with 2-ml Tris-NH_4_Cl buffer solution (the proportion of 0.16 mol/l NH_4_Cl and 0.17 mol/l Tris was 9:1, pH 7.2) to lyse red blood cells. After 5 min of treatment, the splenocyte suspension was replenished to 5 ml with RPMI-1640 medium and then centrifugated at 1,000 rpm for 10 min at 4°C. The precipitated splenocytes of each group were washed twice and adjusted to 5 × 10^6^ cells/ml with 10% FBS RPMI-1640. The splenocyte suspension of each group was planted in a 96-well flat bottom plate in 100-μl aliquots. The cells were respectively introduced by the T cell mitogen (ConA, 4 μg/ml, 100 μl per well, five wells for each group) and the B cell mitogen (LPS, 20 μg/ml, 100 μl per well, five wells for each group). Meanwhile, the wells (saline group) receiving complete RPMI-1640 were regarded as control. The cells were cultured for 48 h at 37°C in a humidified incubator (NAPCO 5410, Precision Scientific Instruments, Buffalo, NY, USA) containing 5% CO_2_ and then cultured at 37°C in the dark for 4 h following the administration of 20 μl MTT (0.5 mg/ml) into each well. After the removal of the suspension, 200 μl of 10% SDS was added to each well to dissolve the formazan, and then cells were cultured for another 12 h under identical conditions. Lymphocyte proliferation activity was detected by absorbance at a wavelength of 570 nm using a microplate reader (Thermo Fisher Scientific Inc., Waltham, MA, USA).

### Analysis of lymphocyte subset

Phenotypic analyses of lymphocytes were performed using a flow cytometer. The splenocytes were incubated with monoclonal antibodies against CD3, CD19, CD4, and CD8 conjugated with different fluorochromes (anti-CD3 (Percp), anti-CD19 (APC), anti-CD4 (PE), and anti-CD8 (FITC)) for 30 min in the dark and then washed three times, resuspended in FACS permeabilizing solution before determination. At least 10,000 cells were analyzed for each Mab staining using a FACScan flow cytometer (Becton Dickinson, Franklin Lakes, NJ, USA).

### Detection of cytokines

Thymocyte suspension was prepared from thymocytes in the RPMI-1640 medium. The suspension of thymocyte and splenocyte was adjusted to 1 × 10^7^ and 2 × 10^7^ cells/ml, respectively, and planted into the 24-well flat-bottom plate (0.5 ml per well). ConA was added to the final concentration of 5 μg/ml to introduce cytokine secretion. The cells were cultured for 48 h at 37°C in a humidified incubator containing 5% CO_2_ at 37°C. The supernatant of each well was collected for cytokine analysis. IL-4 and IFN-γ ELISA kits were used. Briefly, 50-μl samples or standard control were mixed with 50-μl assay diluents and incubated at 37°C for 90 min. After being washed five times, 100-μl antibody-labeled biotin was added to each well. The plate was incubated for 60 min. Following five times of rinsing, a 100-μl substrate solution was added to each well and incubated for 30 min. Finally, a 100-μl stop solution was added to each well, and the colored reaction product was measured at 450 nm on a microplate reader (Thermo Fisher Scientific Inc.).

### The expression level of cytokine analysis by Western blot

Fresh spleens of mouse in each group were stored in ice-cold tubes, and then the total proteins were extracted from the organs. The protein concentration was analyzed using BCA protein assay kit. The proteins in the spleen extracts were separated by 10% SDS-PAGE and electrophoretically transferred onto a polyvinlidene difluoride membrane (Bio-Rad, Hercules, CA, USA). The membranes were blocked with 5% nonfat milk in TBS containing 0.1% Tween 20 at 37°C for 2 h followed by incubation overnight at 4°C with antibodies against IL-12, IFN-γ, IL-4, and TNF-α. β-Actin was taken as the reference protein. The membranes were washed with TBS containing 0.1% Tween 20 and probed with horseradish peroxidase-labeled goat anti-rabbit or anti-mouseIgG. The proteins were detected with enhanced chemiluminescence imaging.

### Statistical analysis

Data were analyzed using the Statistical Package for Social Science (version 19.0; SPSS Inc., IBM, Armonk, NY, USA). The significant difference between groups was analyzed using one-way ANOVA; *P* < 0.05 was considered statistically significant.

## Results and discussion

### Results

#### The characteristic of carbon dots

**Figure 1:**
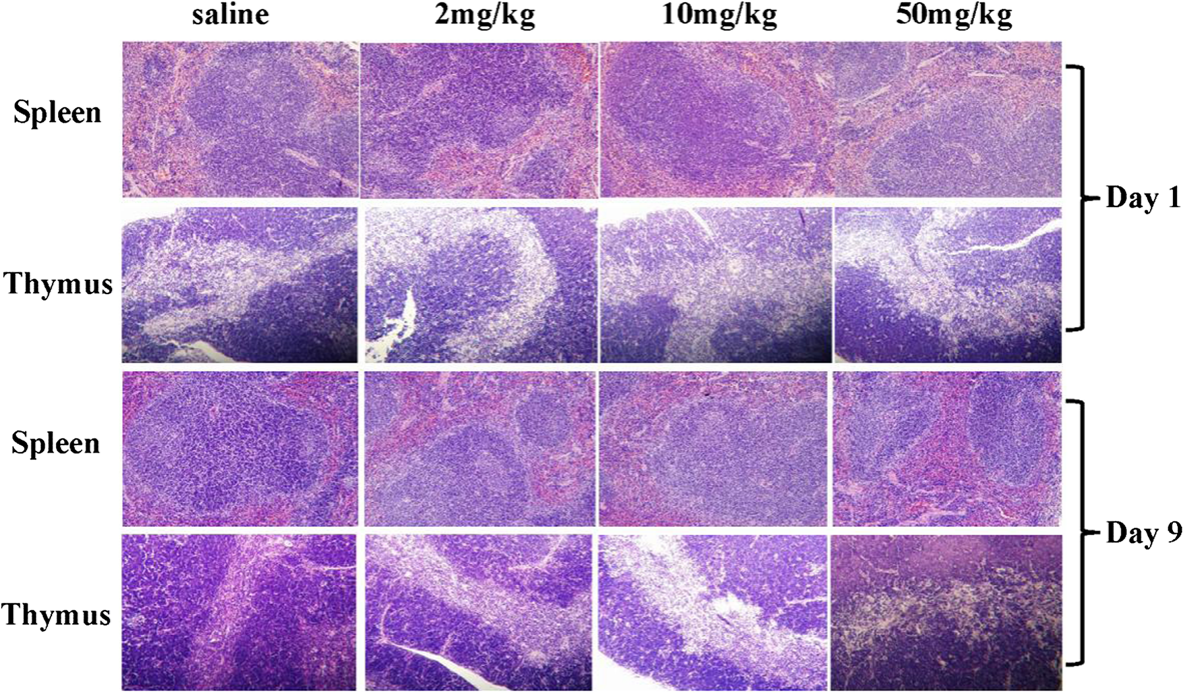
**UV–vis absorption, PL emission spectra, AFM height images, and section analysis of carbon dots.** (**a**) UV–vis absorption of carbon dots-NH_2_. (**b**) Photoluminescence emission spectra of carbon dots with progressively excitation wavelength from 320 to 400 nm in 10-nm increment; inset is the solution illuminated with a UV lamp, (**c**) AFM height images of carbon dots. (**d**) The section analysis of carbon dots.

As shown in Figure 1, the UV–vis absorption spectra of carbon dots and photoluminescence (PL) emission spectra excited by various incident lights are shown in Figure 1a,b, respectively. At an excitation wavelength of 340 nm, a strong emission peak at about 430 nm was observed in the PL emission spectrum of carbon dots. When the excitation wavelength increased from 320 to 400 nm, the emission peak also shifted with a maxima redshift of about 10 nm. The AFM height images and section analysis demonstrated that the diameters of the carbon dots were 3 to 8 nm and the sizes of nanoparticles were spherical and uniform (Figure 1c,d). Due to the existence of carboxyl and hydroxyl groups on the surface of carbon dots, the carbon dots were found to dissolve easily in water and polarity organic solvent (such as ethanol, acetone) but were insolubilized in apolar organic solvent.

#### Organ weight and histological analysis

**Figure 2:**
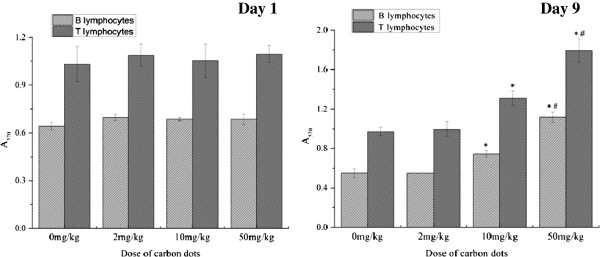
**Histopathological analyses of spleen and thymus of mice.** Mice were injected in the caudal vein with different doses of carbon dots. The samples of spleen and thymus were separated for histopathological analysis. There were no necrosis and hydropic degeneration observed in the splenetic and thymic sections in carbon dot-treated mice both on the first and ninth days post exposure.

BALB/c mice treated with carbon dots appeared healthy, and their body weight gain patterns were similar to those of the control group. At 1 day post exposure, both immune organ (spleens and thymuses) weight coefficients showed no difference between the experimental group and the control group (Table 1). As shown in Figure 2, the structures of the immune organs from the exposed mice were normal. There were no necrosis and hydropic degeneration observed in the splenetic and thymic sections from the exposed mice. On the ninth day after administration, little difference was also found in the weight coefficients and the pathological analysis of immune organs from the carbon dot-treated mice compared with those of the saline control (Table 1; Figure 2). It suggested that carbon dots caused little morphological and histopathological changes in the spleen and thymus.

**Table 1:** Effects of carbon dots on spleen and thymus weight coefficient of BALB/c mice

**Groups**	**Spleen coefficient**		**Thymus coefficient**	
	**1 day**	**9 days**	**1 day**	**9 days**
Saline	0.3616 ± 0.0027	0.9817 ± 0.1343	0.2305 ± 0.0148	0.2598 ± 0.0955
Carbon dots				
2 mg/kg	0.3711 ± 0.0128*	0.8617 ± 0.2637*	0.2092 ± 0.0502*	0.2707 ± 0.0687*
10 mg/kg	0.4020 ± 0.0537*	0.8443 ± 0.0871*	0.2057 ± 0.0328*	0.2793 ± 0.0215*
50 mg/kg	0.4469 ± 0.0846*	0.9927 ± 0.3637*	0.1886 ± 0.0095*	0.2653 ± 0.0398*

The data are presented as mean ± standard deviations, *n* = 5. *P > 0.05 compared with the saline group (control). Significant difference was calculated by one-way ANOVA using SPSS19.0.

#### Splenocyte proliferation

**Figure 3:**
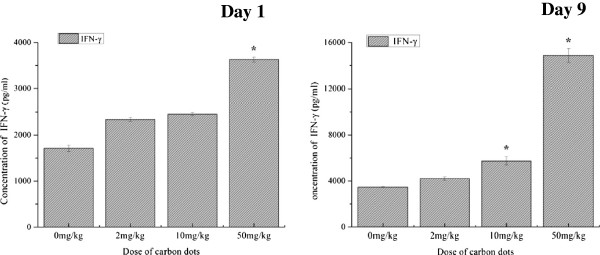
**Influence of carbon dots on splenocyte proliferation of BALB/c mice.** BALB/c mice were injected in the caudal vein with different doses of carbon dots. Spleen samples were separated to prepare splenocytes at 1 or 9 days after the administration. T lymphocytes were introduced by ConA, and B lymphocytes were introduced by LPS. Data are presented as means ± standard deviations, *n* = 5. *P < 0.01 compared with saline group; #*P* < 0.01 compared with lower dose carbon dot-treated group. Significant difference was calculated by one-way ANOVA using SPSS19.0.

On the first day after administration, both B and T lymphocyte proliferations were measured, and the result indicated that carbon dot treatment could not influence the spleen B and T lymphocyte proliferations. However, both T and B lymphocytes were found to have increased and proliferated in the carbon dot-treated groups compared with the saline control group on the ninth day post exposure (*P* < 0.05; Figure 3). Furthermore, the proliferative capacity of lymphocytes was dependent on the dose of carbon dots. The 50-mg/kg administration of carbon dots had a more significant effect on the T lymphocyte proliferation than the 2-mg/kg administration (*P* < 0.05). The B lymphocyte proliferation in mice treated with 50 mg/kg of carbon dots increased significantly compared with the other two groups treated with carbon dots (*P* < 0.05; Figure 3).

#### The proportions of lymphocyte subsets

The percentage of CD3+ and CD19+ represented the relative quantities of T and B lymphocytes, and the percentage of CD4+ and CD8+ explained the proportion of helper T (Th) cells and cytotoxic T (Tc) cells, respectively. Compared with the saline group, only the 50-mg/kg group had a significant percentage of CD19+ (*P* < 0.05; Table 2); all of the three carbon dot-treated groups were found to have a decrease in the ratio of CD4+/CD8+ versus the control group on the first day after administration (*P* < 0.01; Table 3). At 9 days post exposure, a significant increase of the percentage of CD3+ was noticed in the three carbon dot-treated groups versus the control (*P* < 0.01), and the increase of CD19+ percentage was observed in the 2- and 10-mg/kg groups versus the control (*P* < 0.01; Table 4). Furthermore, the ratio of CD3+/CD19+ had an evident increase in all the three carbon dot-treated groups versus the control (*P* < 0.01 for 2 and 50 mg/kg; *P* < 0.05 for 10 mg/kg; Table 4). The percentage of CD19+ in the 10-mg/kg administration groups was higher than that in the other two carbon dot-treated groups (*P* < 0.01; Table 4). Compared with the saline group, the proportion of both CD4+ and CD8+ T lymphocyte subsets was increased in drug-treated groups versus the control (*P* < 0.01; Table 5). However, administration of carbon dots decreased the ratio of CD4+/CD8+, especially for the 2-mg/kg group versus the control (*P* < 0.05; Table 5), whereas there was no difference in the percentage of CD4+ and CD8+ between the administration groups (*P* > 0.05; Table 5).

**Table 2:** **Effects of carbon dots on percentage of CD3**^**+ **^**and CD19**^**+ **^**lymphocytes in spleen of BALB/c mice**

**Groups**	**CD3+ (%)**	**CD19+ (%)**	**CD3+/CD19+**
Saline	36.01 ± 1.62	58.01 ± 1.55	0.62 ± 0.04
Carbon dots			
2 mg/kg	37.44 ± 0.32	57.44 ± 0.55	0.65 ± 0.01
10 mg/kg	35.12 ± 0.39	58.09 ± 0.32	0.60 ± 0.01
50 mg/kg	36.97 ± 1.81**↑	55.81 ± 0.73*↓	0.70 ± 0.02**↑

The effects were recorded 1 day after administration. The data are presented as mean ± standard deviations, *n* = 5. *P < 0.05 and **P < 0.01 compared with the saline group (control). Significant difference was calculated by one-way ANOVA using SPSS19.0.

**Table 3:** Effects of carbon dots on T lymphocyte subsets in spleen of BALB/c mice

**Groups**	**CD4+ (%)**	**CD8+ (%)**	**CD4+/CD8+**
Saline	25.97 ± 0.65	9.94 ± 1.01	2.63 ± 0.21
Carbon dots			
2 mg/kg	24.95 ± 0.20	12.54 ± 0.26**↑	1.99 ± 0.04**↓
10 mg/kg	24.31 ± 0.41**↓	11.00 ± 0.14	2.21 ± 0.05**↓
50 mg/kg	26.51 ± 0.44	12.75 ± 0.12**↑	2.08 ± 0.04**↓

The effects were recorded 1 day after administration. Data are presented as mean ± standard deviations, *n* = 5. **P < 0.01 compared with the saline group (control). Significant difference was calculated by one-way ANOVA using SPSS19.0.

**Table 4:** **Effects of carbon dots on percentage of CD3**^**+ **^**and CD19**^**+ **^**lymphocytes in spleen of BALB/c mice**

**Groups**	**CD3+ (%)**	**CD19+ (%)**	**CD3+/CD19+**
Saline	18.00 ± 1.40	28.74 ± 1.14	0.63 ± 0.02
Carbon dots			
2 mg/kg	26.48 ± 0.52**↑	33.88 ± 0.56**↑	0.78 ± 0.02**↑
10 mg/kg	25.50 ± 0.36**↑	35.95 ± 0.94**↑	0.71 ± 0.03*↑
50 mg/kg	26.68 ± 0.57**↑	29.87 ± 1.07	0.89 ± 0.05**↑

The effects were recorded 9 days after administration. Data are presented as mean ± standard deviations, *n* = 5. *P < 0.05 and **P < 0.01 compared with the saline group (control). Significant difference was calculated by one-way ANOVA using SPSS19.0.

**Table 5:** Effects of carbon dots on T lymphocyte subsets in spleen of BALB/c mice

**Groups**	**CD4+ (%)**	**CD8+ (%)**	**CD4+/CD8+**
Saline	10.85 ± 1.15	5.47 ± 0.62	1.99 ± 0.17
Carbon dots			
2 mg/kg	16.05 ± 0.24**↑	9.89 ± 0.40**↑	1.63 ± 0.09*↓
10 mg/kg	15.77 ± 0.59**↑	9.16 ± 0.28**↑	1.73 ± 0.12
50 mg/kg	16.56 ± 0.28**↑	9.65 ± 0.44**↑	1.72 ± 0.05

The effects were recorded 9 days after administration. The data are presented as mean ± standard deviations, *n* = 5. *P < 0.05 and **P < 0.01 compared with the saline group (control). Significant difference was calculated by one-way ANOVA using SPSS19.0.

#### Influence on cytokine production

**Figure 4:**
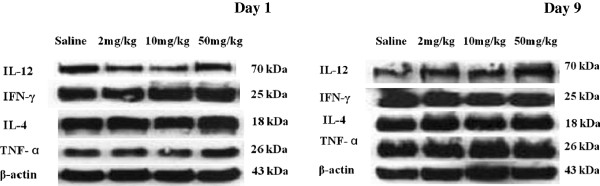
**Concentration of cytokine INF-γ in splenocyte suspension.** The levels of INF-γ were measured quantitatively using IFN-γ ELISA kit. Data are presented as means ± standard deviations, *n* = 5. *P < 0.01 compared with saline control. Significant difference was calculated by one-way ANOVA using SPSS19.0.

Cytokines IFN-γ and IL-4 levels in sera of mice were not detected in the preliminary experiment (data were not shown). Therefore, splenocyte and thymocyte suspensions were used to assay the production of cytokines. At 1 day post treatment, the secretion of IFN-γ promoted significantly in the 50-mg/kg group (*P* < 0.01; Figure 4), and a slightly increased level of IFN-γ was also found in the other two treated groups versus the saline group (*P* > 0.05; Figure 4). On the ninth day after administration, the result of cytokine assay indicated that carbon dot treatment could significantly increase the secretion of IFN-γ and displayed as dose dependent (*P* < 0.01, for 10- and 50-mg/kg administration groups versus saline control; Figure 4). There was a significant difference in the production of IFN-γ between the carbon dot administration groups (*P* < 0.01). However, the secretion of IL-4 in thymocyte suspensions was not detected in all experimental groups both at 1 and 9 days after administration (data were not presented).

#### Effect on the expression level of the cytokines

**Figure 5:**
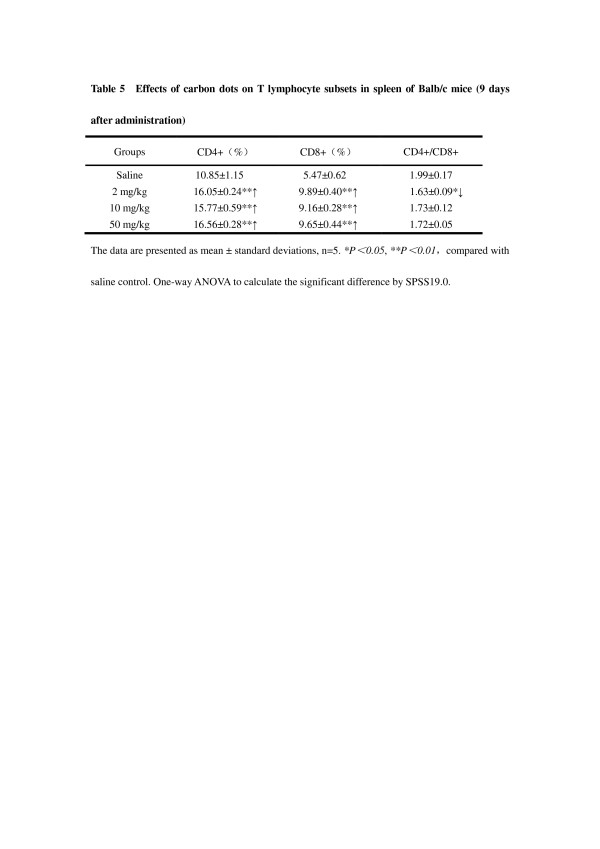
**IL-12, IFN-γ, IL-4, and TNF-α in spleens of mice treated with carbon dots.** Western blot was used to measure the levels of cytokines. Compared with the saline group, the expression levels of four cytokines did not have any obvious change in the three carbon dot-treated groups both on the first and ninth days post exposure.

Cytokines play an important role in cellular immunity. To clarify the possible mechanism of the effects of carbon dots to the immune system in mice, the expression levels of IL-12, IFN-γ, IL-4, and TNF-α in the spleens of mice treated with carbon dots were detected by Western blot. Compared with the saline group, the expression levels of four cytokines did not have any obvious change in the three carbon dot administration groups both on the first and ninth days after administration (Figure 5).

## Discussion

B and T lymphocytes, which play an important role in the process of adaptive immunity, are the central cells of the immune system. Both of them are resting cells in the G_0_ phase of the cell cycle when they have not interacted with antigens. Once stimulated by certain mitogens, these cells would be activated into the cell cycle (by progressing from G_0_ into G_1_ and subsequently into S, G_2_, and M) and promoted to proliferate and differentiate. Thus, the proliferation of lymphocytes following exposure to mitogenic stimuli is an important methodology for the assessment of cell-mediated immunity [16]. In the present study, we investigated the influence of carbon dots on lymphoproliferation in the spleen following exposure to the B cell mitogen (LPS) and T cell mitogen (ConA). As the results showed, splenic lymphocytes had little increase in proliferation in the carbon dot groups at 1 day post exposure. However, both B and T lymphocyte proliferation in treated groups increased significantly in a dose-dependent manner on the ninth day after administration.

B and T lymphocytes can be distinguished by the presence of either CD3 or CD19 membrane glycoproteins on their surfaces; thus, the number of T and B lymphocytes can be approximated by assaying the percentage of CD3+ and CD19+. Also, the subsets of T lymphocytes can be distinguished by the presence of CD4 and CD8. At 1 day after administration, the percentage of CD3+ and the ratio of CD3+/CD19+ have an evident increase only in the 50-mg/kg group. Nevertheless, the three administered groups were detected an obvious increase in the percentage of CD3+ and the ratio of CD3+/CD19+ without a dose-dependent relationship. The result of higher ratios of CD3+/CD19+ in all of the three carbon dot-treated groups indicated that the proliferation of T lymphocytes was more significant than that of B lymphocytes in peripheral lymphocytes under the administration of carbon dots, which coincided with the results of splenocyte proliferation.

The two major subpopulations of T lymphocytes are Th cells and Tc cells. In general, CD4+ cells act as helper cells and CD8+ cells act as cytotoxic cells. The Th cells can also be defined as two major functional subpopulations, Th1 and Th2 cells. The Th1 response produces cytokines (IFN-γ, TNF-β, etc.) that support inflammation and activate mainly certain T cells and macrophages, whereas the Th2 response secretes cytokines (IL-4, IL-5, etc.) which activate mainly B cells and immune responses that depend upon antibodies [17,18]. The Tc cells can recognize antigens combined with class I MHC in the presence of appropriate cytokines (IFN-γ) and give rise to cytotoxic T cells, which display cytotoxic ability. Several studies have addressed the influence of nanoparticles on Th1 and Th2 responses. It is reported that some small engineered nanoparticles such as 80- and 100-nm nanoemulsions, 95- and 112-nm PEG-PHDA nanoparticles, and 123-nm dendrosome, could induce the Th1 response [19]. We observed that carbon dots could promote the percentage of CD8+ and decrease the ration of CD4+/CD8+. Nevertheless, both the percentages of CD8+ and CD4+ had a significant increase without a dose-dependent relationship at 9 days after administration, and the ration of CD4+/CD8+ decreased only in the 2-mg/kg group. The levels of IFN-γ also had a significant increase in the carbon dot-treated groups. From these results, we presume that the main modulator pathway of carbon dots was to activate the Th1 cells. The Th1 cells secreted IFN-γ cytokines, which played an important role in the activation of the proliferation and differentiation of the Tc cells (CD8+ T cell), and then the percentage of CD8+ increased, and the ratios of CD4+/CD8+ declined. The IFN-γ cytokines could also be produced by Tc cells, which were dedicated to the increase of the levels of IFN-γ. On the other hand, the production of IL-4 cytokines was hardly to be detected both in the blood serum and the supernatant of induced lymphocytes, indicating that carbon dots, at the treated dose, could not induce the response of Th2 cells, which play an important role in the activation process of humoral immunity. The results of Western blot did not show any changes in the cytokine levels in the peripheral immune organs, which is in accordance with the result of the weight coefficients and histological analysis of the spleen and thymus.

## Conclusions

A delicate balance between innate and adaptive immunity is required for efficient functioning of the immune system. This balance is important in cancer immunity, immune response against pathogens, and avoiding hypersensitivity reactions [20]. In this study, we have demonstrated that carbon dots could adjust the immune function of BALB/c mice by inducing Th1 and Tc responses. However, these effects were not enough to induce the morphological change of immune organs. The mechanism by which carbon dots modulate the immune system remains unclear. More systematic and profound studies are needed, and the pertinent testing guidelines for immunological evaluation of nanoparticles need to be formulated quickly.

## Competing interests

The authors declare that they have no competing interests.

## Authors’ contributions

ZCG, ND, and PYJ carried out the main experiments. XNZ, JHW, and YGZ designed and participated in the animal experiments. GXS synthesized and evaluated the carbon dots in this research. GXS, YXW, and DXC participated in the design and coordination of this study. All authors read and approved the final manuscript.
